# Removal of the magnetic sphincter augmentation device: an assessment of etiology, clinical presentation, and management

**DOI:** 10.1007/s00464-023-09878-y

**Published:** 2023-01-23

**Authors:** Sven Eriksson, Katrin Schwameis, Shahin Ayazi, Toshitaka Hoppo, Ping Zheng, Blair A. Jobe

**Affiliations:** 1 Esophageal Institute, Allegheny Health Network, Pittsburgh, PA USA; 2 Department of Surgery, Drexel University, Philadelphia, PA USA; 3 Esophageal Institute, Allegheny Health Network, 4815 Liberty Avenue, Suite 439, Pittsburgh, PA 15224 USA

**Keywords:** Magnetic sphincter augmentation, Device removal, Gastroesophageal reflux disease, Symptom recurrence, Dysphagia

## Abstract

**Background:**

Magnetic sphincter augmentation (MSA) erosion, disruption or displacement clearly requires device removal. However, up to 5.5% of patients without anatomical failure require removal for dysphagia or recurrent GERD symptoms. Studies characterizing these patients or their management are limited. We aimed to characterize these patients, compare their outcomes, and determine the necessity for further reflux surgery.

**Methods:**

This is a retrospective review of 777 patients who underwent MSA at our institution between 2013 and 2021. Patients who underwent device removal for persistent dysphagia or recurrent GERD symptoms were included. Demographic, clinical, objective testing, and quality of life data obtained preoperatively, after implantation and following removal were compared between removal for dysphagia and GERD groups. Sub-analyses were performed comparing outcomes with and without an anti-reflux surgery (ARS) at the time of removal.

**Results:**

A total of 40 (5.1%) patients underwent device removal, 31 (77.5%) for dysphagia and 9 (22.5%) for GERD. After implantation, dysphagia patients had less heartburn (12.9-vs-77.7%, *p* = 0.0005) less regurgitation (16.1-vs-55.5%, *p* = 0.0286), and more pH-normalization (91.7-vs-33.3%, *p* = 0.0158). Removal without ARS was performed in 5 (55.6%) GERD and 22 (71.0%) dysphagia patients. Removal for dysphagia patients had more complete symptom resolution (63.6-vs-0.0%, *p* = 0.0159), freedom from PPIs (81.8-vs-0.0%, *p* = 0.0016) and pH-normalization (77.8-vs-0.0%, *p* = 0.0455).

Patients who underwent removal for dysphagia had comparable symptom resolution (*p* = 0.6770, freedom from PPI (*p* = 0.3841) and pH-normalization (*p* = 0.2534) with or without ARS. Those who refused ARS with removal for GERD had more heartburn (100.0%-vs-25.0%, *p* = 0.0476), regurgitation (80.0%-vs-0.0%, *p* = 0.0476) and PPI use (75.0%-vs-0.0%, *p* = 0.0476).

**Conclusions:**

MSA removal outcomes are dependent on the indication for removal. Removal for dysphagia yields excellent outcomes regardless of anti-reflux surgery. Patients with persistent GERD had worse outcomes on all measures without ARS. We propose a tailored approach to MSA removal-based indication for removal.

**Graphical abstract:**

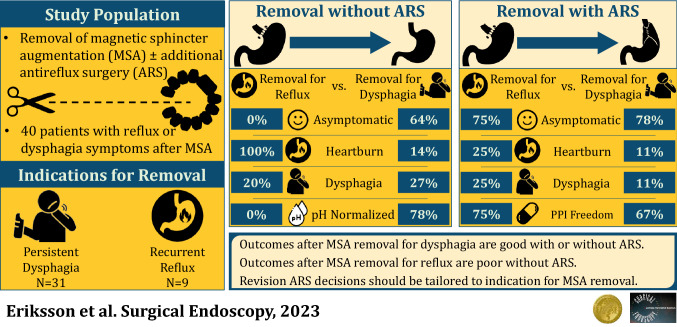

Magnetic sphincter augmentation (MSA) is a highly effective anti-reflux surgery (ARS) in patients with gastroesophageal reflux disease (GERD) [1]. Studies have shown significant improvement in GERD-health-related quality of life (HRQL) score and high rates of freedom from antisecretory medication and pH normalization following this procedure. [2–4] The advantage of the MSA, compared to Nissen fundoplication, is that it is reversible, technically standardized, largely preserves gastric anatomy and has lower rates of side effects such as gas-bloat syndrome [5]. However, despite its excellent efficacy and superior side effect profile, dysphagia remains the most common complaint after MSA [6]. Up to 31% of patients with persistent post-operative dysphagia require at least one endoscopic dilation [7]. Although, dysphagia can be managed effectively with diet and dilation in the majority of patients, some will require device removal [7]. Published removal rates range from 0.5 to 8.3% of patients, with persistent dysphagia or recurrent GERD symptoms often listed as the most common indications for removal. [8–12] The management for anatomic failures such as recurrent hernia, erosion, device displacement or device disruption is straightforward: surgical revision. However, the approach to the symptomatic patient with anatomic integrity is less clear. This population and their device removal management regimens have not been well described in the literature.

The current study was designed to characterize the groups of patients who required device removal for recurrent GERD symptoms or persistent dysphagia despite no objective evidence of device erosion, displacement or disruption. The clinical outcomes after device removal, and the necessity for further treatment of post-removal GERD symptoms were assessed. Based on these results, we proposed a treatment paradigm to assist with management of patients who are being considered for device removal.

## Material and methods

### Study design and population

This was a retrospective review of prospectively collected data from patients who underwent magnetic sphincter augmentation (MSA) between 2013 and 2021 at the Esophageal Institute, Allegheny Health Network (AHN) (Pittsburgh, PA). Inclusion criteria were patients with an age of 18 years or older with no history of foregut surgery prior to MSA, who underwent device removal for either persistent dysphagia or recurrent GERD symptoms and had at least 6 months post-removal follow-up. This study was reviewed and approved by the Institutional Review Board of the AHN (IRB 2021–259).

### Evaluation prior to MSA

All patients underwent complete foregut evaluation prior to index surgery, consisting of a detailed clinical examination, esophagogastroduodenoscopy (EGD), video-esophagram and esophageal physiology testing such as pH monitoring and manometry. Additionally, patients were asked to complete standardized questionnaires including the GERD health-related quality of life (GERD-HRQL) survey [13]. The GERD-HRQL objectively assesses overall patient satisfaction and severity of reflux symptoms using a 0 to 5 rating scale. Esophageal motility was assessed by high-resolution manometry (HRM). Once off proton pump inhibitors for 10 days, patients underwent ambulatory wireless 48-h Bravo® pH monitoring (Medtronics, Shoreview, MN). [14] A DeMeester score > 14.7 was considered abnormal acid exposure to the distal esophagus.

### Surgical technique for MSA

The LINX® reflux management system (Ethicon, Johnson & Johnson; Shoreview, MN), consists of interlinked magnetic titanium beads and features a Roman Arch design assuring non-compressing device closure [15]. Its dynamic design ensures that the esophageal range of motion is not limited.

All procedures were performed laparoscopically by experienced foregut surgeons using standardized surgical techniques as previously described. The procedure consists of complete mediastinal dissection to obtain adequate intraabdominal esophageal length (≥ 2 cm), posterior crural closure and device placement at the level of the gastroesophageal junction with the posterior vagus nerve trunk located on the outside of the device. This approach was used in all patients regardless of whether there was a hiatal hernia. Many patients have transverse widening of the hiatal opening with minimal axial displacement and our approach is focused on restoring the crural contribution of the antireflux barrier during device placement. Intraoperative EGD was performed in order to assist in identifying the anatomic GEJ and to assess device position.

A solid “LINX” diet was started on the day of surgery to avoid development of dysphagia due to formation of scar tissue surrounding the device. Patients were encouraged to eat small portions of solid food every hour while awake for the first two postoperative months. Discharge from hospital occurred on the day of surgery in majority of patients.

### Post device implantation assessment

Postoperative follow-up visits were routinely scheduled at 2 weeks, 6 weeks, 6 months and annually after MSA. Patients were assessed for clinical resolution of their symptoms and freedom from anti-secretory medications. At our institution, the typical post-operative assessment regimen after MSA included 6 month and annual GERD-HRQL assessments, and at one-year postoperative patients were approached to repeat their esophageal physiology testing. Patients who reported dysphagia within the first 8 weeks after surgery were initially managed with dietary modification and observed, as the majority of early post-operative dysphagia resolves without intervention. If patients continued to complain of persistent dysphagia as defined by a score > 3 on the GERD-HRQL “difficulty swallowing” item 8 weeks after surgery, despite following dietary recommendations, they were considered for fluoroscopically guided sequential through-the-scope balloon dilation of the magnetically augmented lower esophageal sphincter. Patients who underwent dilation received a seven-day course of twice daily 20 mg prednisone. Patients who failed to respond to three dilations or had recurrent reflux symptoms were considered for device removal.

### Surgical technique of device removal

Device removal was performed laparoscopically by the implanting surgeon in most cases. The outer fibrous capsule surrounding the LINX device was opened with Harmonic Shears (Ethicon, Johnson and Johnson, Raritan, NJ) or laparoscopic scissors and each anterior bead was sequentially freed of its capsular attachments. Once sufficient exposure was achieved, the device was disconnected either by disarticulating the clasp or cutting the interconnecting wire with Harmonic shears. Further capsular dissection followed, systematically freeing the posterior beads while applying gentle traction until the device was free. Whenever possible, the device was removed in one piece through the abdominal port. The inner capsule, in contact with the esophagus and anterior vagus nerve, and the small portion of outer capsule in the region of the posterior vagus nerve were left unviolated. After removal from the body, the beads were counted and the surgical sites and surrounding tissues were carefully inspected for injury. Upper endoscopy was performed to confirm no perforation or bleeding. Hiatal hernia repair and/or further anti-reflux procedures were then performed, if indicated.

### Post LINX® removal assessment and outcomes

After device removal, patients were assessed using a similar post-operative clinic schedule as post-implantation patients. Patients were divided into two groups based on whether persistent post-operative dysphagia (removal for dysphagia group) or persistent or recurrent reflux symptoms (removal for GERD group) was the indication for device removal. Baseline demographics and objective testing results were compared between two groups. Additionally, primary GERD symptoms (heartburn, regurgitation, dysphagia), PPI use, and objective esophageal physiology testing at baseline, post-implantation, and post-removal were compared for each group.

The possibility of performing an additional ARS was discussed with all patients prior to device removal. All patients with objective evidence of recurrent GERD were recommended ARS at the time of removal. The final decision as to whether or not to proceed with an additional ARS was determined by shared decision making, factoring in surgeon recommendation and patient preference. Sub-analyses comparing those who underwent additional ARS at the time of device removal to those who refused ARS were performed.

### Statistical analysis

Data are described as median (interquartile range), mean (standard deviation), or frequency (percent), where appropriate. Statistical analysis was performed using non-parametric tests. Categorical variables were assessed using the Fisher exact test and continuous data using the Wilcoxon Rank test or Kruskal–Wallis test, as appropriate. A *p* value < 0.05 was considered to be statistically significant. All statistical analyses were performed using SAS software (version 9.4, SAS Institute).

## Results

A total of 777 patients underwent MSA between 2013 and 2021 at our institution. Of these, 40 (5.1%) patients had device removal for recurrent GERD or persistent dysphagia. There were 9 (22%) patients in removal for GERD group and 31 (77%) in removal for dysphagia group. Baseline demographic and clinical characteristics are summarized in Table 1. The total population was 71% female with a median (IQR) BMI of 29.1 (22.5–31.9). Demographics and baseline clinical characteristics were comparable between groups. Pre-operative objective testing results are summarized in Table 2. Patients in the removal for GERD group had significantly more LA grade C or D esophagitis than patients in the removal for dysphagia group (22.2 vs. 0.0%, *p* = 0.0462). Patients who underwent removal for dysphagia were more frequently implanted with a smaller sized device (13 or 14 beads) than the removal for GERD group, but this difference was not significant (87.1 vs. 55.6%, *p* = 0.0594).

**Table 1:** Baseline demographic and clinical characteristics

	Total population (*n* = 40)	Recurrent GERD (*n* = 9)	Dysphagia (*n* = 31)	*p*-value
Age, median (IQR), years	52.0 (37.0–62.5)	37.0 (31.0–56.0)	53 (45.0–63.0)	0.1084
Sex				
Male, *n* (%)	9 (29.0%)	4 (44.4%)	5 (16.1%)	0.1680
Female, *n* (%)	31 (71.0%)	5 (55.6%)	26 (83.9%)	
BMI, median (IQR), kg/m^2^	29.1 (22.5–31.9)	30.3 (24.0–31.3)	27.7 (20.9–32.0)	0.7095
BMI ≥ 30, *n* (%)	19 (47.5%)	5 (55.6%)	14 (45.2%)	0.7116
GERD Symptoms				
Typical, *n* (%)	24 (60.0%)	7 (77.8%)	17 (54.8%)	0.2717
Atypical, *n* (%)	16 (40.0%)	2 (22.2%)	14 (45.2%)	
GERD-HRQL, Median (IQR), Total Score	39.0 (17.0–52.0)	46.0 (23.0–53.5)	39.0 (14.5–49.5)	0.7164
Dysphagia, *n* (%)	7 (17.5%)	0 (0.0%)	7 (22.6%)	0.1747

**Table 2:** Pre-operative objective testing

	Recurrent GERD (*n* = 9)	Dysphagia (*n* = 31)	*p*-value
Hiatal hernia, *n* (%)	6 (66.7%)	28 (90.3%)	0.1147
Hiatal hernia > 3 cm, *n* (%)	0 (0.0%)	1 (3.2%)	1.0000
Esophagitis, *n* (%)	5 (55.6%)	9 (29.0%)	0.2338
LA C + D, *n* (%)	2 (22.2%)	0 (0.0%)	0.0462
History of Barrett’s, *n* (%)	1 (11.1%)	3 (9.7%)	1.0000
DeMeester Score, Median (IQR)	44.4 (18.7–68.0)	20.0 (15.3–29.8)	0.1770
DeMeester Score > 14.7, *n* (%)	7 (77.8%)	23 (74.2%)	1.0000
Manometric features, median (IQR)			
LES length	2.8 (2.2–3.1)	3.2 (2.6–4.0)	0.1355
Intrabdominal length	1.1 (0.0–1.4)	1.5 (0.1–2.5)	0.2184
Resting pressure	25.6 (12.6–28.9)	29.7 (21.5–33.4)	0.1521
Residual pressure	10.0 (6.7–10.3)	9.7 (7.1–11.0)	0.8759
Distal contractile integral	1764 (999.4–3166)	2019 (1518–3073)	0.6288
Distal wave amplitude	95.2 (57.5–123.8)	93.3 (78.8–119.3)	0.8342
Percent peristalsis	95.0 (85.0–100.0)	100.0 (90.0–100.0)	0.6520
Incomplete bolus clearance	5.0 (0.0–70.0)	0.0 (0.0–30.0)	0.3712

### Post-implantation outcomes

Subjective and objective surgical outcomes after MSA are summarized in Table 3. Among the patients in the removal for dysphagia group, 29% reported either heartburn or regurgitation. However, they had significantly lower rates of heartburn (12.9 vs. 77.7%, *p* = 0.0005) and regurgitation (16.1 vs. 55.5%, *p* = 0.0286) compared to patients in the removal for GERD group. Dysphagia was present in 44.4% of removal for GERD patients and 100.0% of removal for dysphagia patients (*p* = 0.0002). There was a significant reduction in the median GERD-HRQL total scores after device implantation in the removal for dysphagia group (39.0 to 17.5, *p* = 0.0465), but not in the removal for GERD group (*p* = 0.2934). Normalization of distal esophageal pH was achieved by 91.7% in the removal for dysphagia group as opposed to only 33.3% in the removal for GERD group (*p* = 0.0158). Manometry revealed that patients in the removal for dysphagia group had higher median residual pressures (19.2 vs. 14.3, *p* = 0.156) and rates of incomplete bolus clearance (40.0 vs. 0.0%, *p* = 0.0452) compared to patients in the removal for GERD group.

**Table 3:** Post-implant clinical and objective findings

	Recurrent GERD (*n* = 9)	Dysphagia (*n* = 31)	*p*-value
GERD symptoms			
Heartburn, * n* (%)	7 (77.8%)	4 (12.9%)	0.0005
Regurgitation, * n* (%)	5 (55.6%)	5 (16.1%)	0.0286
Dysphagia, * n* (%)	4 (44.4%)	31 (100%)	0.0002
GERD-HRQL score, median (IQR)	18 (6–43.8)	17.5 (13–27.5)	0.7615
Freedom from PPI, * n* (%)	6 (75.0%)	17 (85.0%)	0.6056
Satisfaction, * n* (%)	3 (37.5%)	8 (40.0%)	1.0000
DeMeester score, median (IQR)*	20.8 (9.2–42.6)	6.5 (1.7–11.7)	0.0329
pH Normalization, * n* (%)*	3 (33.3%)	11 (91.7%)	0.0158
Manometric features, median (IQR)†			
LES Length	2.9 (2.5–3.4)	3.3 (2.5–3.8)	0.4185
Intra-abdominal length	1.3 (0.6–1.9)	2.3 (1.5–2.9)	0.1476
Resting pressure	25.9 (23.8–31.8)	31.1 (25.1–36.7)	0.2664
Residual pressure	14.3 (11.0–18.7)	19.2 (17.5–23.2)	0.0156
Distal contractile integral	1888 (1148–3294)	2144 (1461–4422)	0.6027
Distal wave amplitude	88.4 (70.8–125.5)	78.0 (47.2–98.1)	0.4665
Percent peristalsis	100.0 (100.0–100.0)	95.0 (50.0–100.0)	0.0612
Incomplete bolus clearance	0.0 (0.0–10.0)	40.0 (0.0–60.0)	0.0452

*12 Dysphagia group patients underwent post-implant Bravo

†21 Dysphagia group patients underwent post-implant manometry

### Removal outcomes

Device removal was performed at a median (IQR) of 23.7 (13.0–27.2) months in the removal for GERD group and 10.6 (5.4–16.7) months in the removal for dysphagia group (*p* = 0.0951). Post-removal follow-up assessments were performed at a median (IQR) of 11.7 (5.0–22.8) months.

There were 5 (55.6%) patients in the removal for GERD group and 22 (71.0%) patients in the removal for dysphagia group that underwent device removal without additional ARS. Clinical and objective outcomes following device removal for these patients are summarized in Table 4. Complete resolution of the typical GERD symptoms was achieved by 63.6% of removal for dysphagia and 0.0% of removal for GERD patients (*p* = 0.0159). Patients who underwent removal for GERD had significantly higher rates of heartburn (100.0 vs. 13.6%, *p* = 0.0007) and regurgitation (80.0 vs 13.6%, *p* = 0.0089). Rates of post-removal dysphagia were comparable between groups (*p* = 1.0000). The removal for dysphagia group was significantly more likely to achieve freedom from anti-secretory medications (81.8 vs. 0.0%, *p* = 0.0016). In a subset of 12 patients (3 removal for GERD; 9 removal for dysphagia) who underwent post-removal pH-monitoring, patients who underwent removal for dysphagia were significantly more likely to achieve pH normalization (77.8 vs. 0.0%, *p* = 0.0455).

**Table 4:** Post-removal without ARS clinical and objective findings

	Recurrent GERD (*n* = 5)	Dysphagia (*n* = 22)	*p*-value
GERD symptoms			
Complete resolution, *n* (%)	0 (0.0%)	14 (63.6%)	0.0159
Heartburn, *n* (%)	5 (100.0%)	3 (13.6%)	0.0007
Regurgitation, *n* (%)	4 (80.0%)	3 (13.6%)	0.0089
Dysphagia, *n* (%)	1 (20.0%)	6 (27.3%)	1.0000
Freedom from PPI, *n* (%)	0 (0.0%)	18 (81.8%)	0.0016
DeMeester score, median (IQR)*	45.4 (20.6–126)	6.4 (3.5–20.1)	0.0398
pH normalization, *n* (%)*	0 (0.0%)	7 (77.8%)	0.0455

*3 GERD and 9 Dysphagia group patients underwent post-removal Bravo

### Impact of ARS at the time of removal

ARS was performed in 4 (44.4%) patients who underwent removal for GERD and 9 (29.0%) patients who underwent removal for dysphagia. The difference in symptomatic outcomes between groups who underwent device removal with and without additional ARS at the time of removal are shown in Table 5. Compared to patients who underwent ARS at removal, patients in the removal for GERD group who refused ARS had significantly lower rates of complete typical GERD symptom resolution (0.0 vs. 75.0%, *p* = 0.0476), due to higher rates of heartburn (100.0 vs 25.0%, *p* = 0.0476) and regurgitation (80.0 vs 0.0%, *p* = 0.0476). All and only patients who achieved complete symptom resolution achieved freedom from PPI in the removal for GERD group. ARS at the time of removal for dysphagia did not significantly impact post-removal symptoms or freedom from PPIs.

**Table 5:** Impact of ARS at removal on post-removal symptoms

		Removal with ARS	Removal without ARS	*p*-value
		(*n* = 4)	(*n* = 5)	
Removal for recurrent GERD	Complete resolution, *n* (%)	3 (75.0%)	0 (0.0%)	0.0476
	Heartburn, *n* (%)	1 (25.0%)	5 (100.0%)	0.0476
	Regurgitation, *n* (%)	0 (0.0%)	4 (80.0%)	0.0476
	Dysphagia, *n* (%)	1 (25.0%)	1 (20.0%)	1.0000
	Freedom from PPI, *n* (%)	3 (75.0%)	0 (0.0%)	0.0476
		(*n* = 9)	(*n* = 22)	
Removal for persistent dysphagia	Complete resolution, *n* (%)	7 (77.8%)	14 (63.6%)	0.6770
	Heartburn, *n* (%)	1 (11.1%)	3 (13.6%)	1.0000
	Regurgitation, *n* (%)	0 (0.0%)	3 (13.6%)	0.5375
	Dysphagia, *n* (%)	1 (11.1%)	6 (27.3%)	0.6395
	Freedom from PPI, *n* (%)	6 (66.7%)	18 (81.8%)	0.3841

In the removal for dysphagia group, post-removal pH monitoring was performed in a subset of 9 patients who underwent removal without ARS and 5 patients who received ARS at the time of removal. Median (IQR) DeMeester score in the removal for dysphagia with ARS group was 2.1 (0.8–6.3) and pH normalization was achieved by 5 (100.0%) patients, compared to a DeMeester score of 6.4 (3.9–13.7) and normalization rate of 7(77.8%) in the removal for dysphagia without ARS group. These DeMeester scores and pH normalization rates were not significantly different (*p* = 0.0605 & *p* = 0.2534, respectfully).

### Dysphagia after removal

**Fig. 1:**
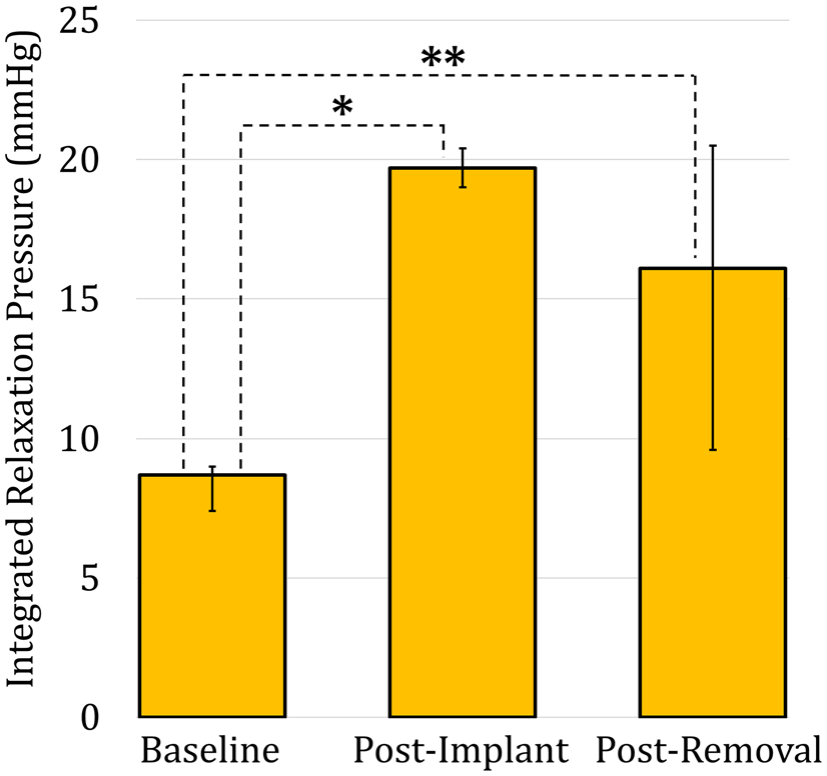
Median and interquartile range (error bars) of the integrated relaxation pressures at baseline, post-implantation, and post-removal among 7 patients with persistent dysphagia after device removal. There was a significant increase from baseline to post-implant (*p* = 0.0015) and baseline to post-removal (*p* = 0.0025)

There were 7 (22.6%) patients in the removal for dysphagia group who continued to report dysphagia after device removal and underwent post-removal manometry**.** Analysis of their baseline, post-implant, and post-removal manometry characteristics was performed. There was a significant difference between the median (IQR) baseline and post-implant IRP [8.7 (8.4–10.0) vs. 19.7 (19.0–20.4), *p* = 0.0015], and the median baseline and post-removal IRP [8.7 (8.4–10.0) vs. 16.1 (11.7–22.6), *p* = 0.0025], but not between the post-implant and post-removal IRP (*p* = 0.2691) (Fig. 1). Additionally, there was a significant increase in median (IQR) percent incomplete bolus clearance from a baseline of 0.0% (0.0–20.0) to 70.0% (40.0–70.0) after implant (*p* = 0.0087), which then significantly decreased to 10.0% (0.0–30.0) after removal (*p* = 0.0465). There was no significant difference between baseline and post-removal bolus clearance (*p* = 0.5265). No other manometric features were significantly different.

## Discussion

The goal of any anti-reflux surgery is to prevent retrograde flow of gastric contents by restoring the competency of the gastro-esophageal junction (GEJ). If postoperative resistance at the GEJ is too high, both retrograde and anterograde flow stops, and the patient suffers from dysphagia [16, 17]. If the GEJ competency has not been restored adequately, the surgery is ineffective and reflux symptoms persist [17]. The ideal management of these postoperative complications should target the underlying mechanism of failure. In the present study, we reported our experience with device removal after MSA due to the complications of recurrent GERD symptoms and persistent dysphagia. Our major finding was that clinical outcomes were dependent on the indication for device removal. Patients who underwent removal for dysphagia had higher rates of heartburn resolution, regurgitation resolution and pH normalization both after implant and removal. Additionally, these patients had comparatively excellent outcomes with or without ARS at the time of removal. By contrast, patients with recurrent GERD symptoms after MSA who refused ARS at the time of removal had significantly worse outcomes, with all patients failing to achieve complete symptom resolution, freedom from PPIs or pH normalization. These findings suggest that surgical management should be tailored to the indication for device removal.

Heartburn, regurgitation and abnormal esophageal acid exposure significantly improved after device implantation in the removal for dysphagia group, suggesting an adequate increase in EGJ resistance, preventing reflux. Similarly, previous studies of dysphagia after fundoplication have shown that reflux symptom control is not diminished by postoperative dysphagia. [18] Studies of patients with dysphagia after MSA have even shown higher rates of pH normalization than those without dysphagia. [19] Further studies have shown that as mean pressure across the GEJ rises, so does severity of dysphagia. [20] These findings suggest that persistent dysphagia after MSA is the result of a supra-competent LES that prevents both retrograde and anterograde flow.

Early dysphagia following MSA is common, but usually self-limiting. [19] Dietary exercises consisting of small bites of solid food every waking hour for the first several weeks after surgery maintains a degree of elasticity at the GEJ and prevents stricture as scar tissue forms and contracts [11]. Early dilation after MSA can trigger an inflammatory reaction in the soft tissue surrounding the device, exacerbating scarification, and should be avoided. [21] Dysphagia lasting more than 8 weeks occurs in up to 19% of cases, and may correspond with the development of a robust capsule around the MSA [22]. Therefore, at our institution consideration for dilation is delayed until at least 8-weeks after MSA. Following dilation under fluoroscopic guidance patients are prescribed a seven-day course of twice daily 20 mg prednisone and dietary modifications to minimize the inflammatory response. After three endoscopic dilations without durable improvement, device removal should be considered.

**Fig. 2:**
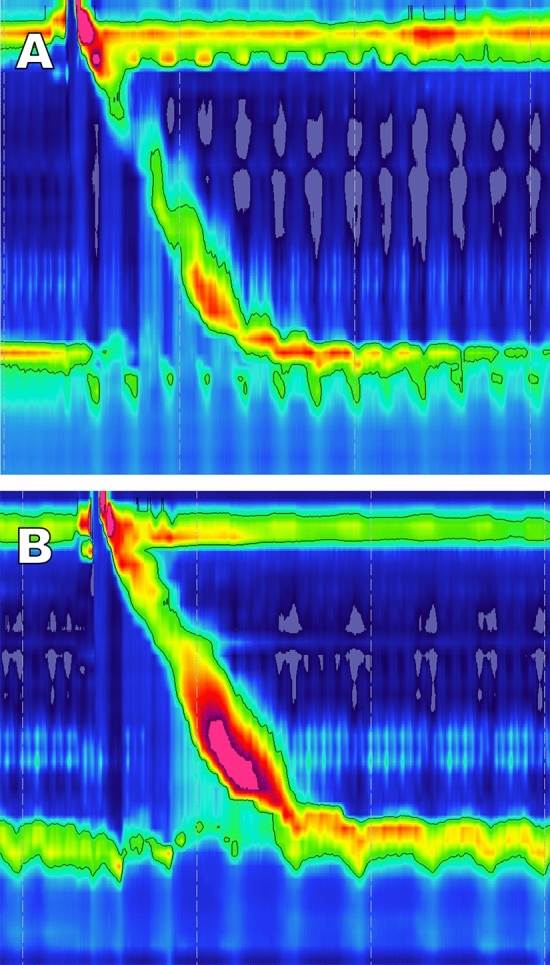
HRM topographic plot before (**A**) and after MSA implantation (**B**), but before removal in a patient who subsequently underwent device removal for dysphagia. At baseline (**A**) the patient has adequate distal contractile integral (DCI) with complete bolus clearance (not shown). There is complete LES relaxation and evidence of some degree of crural-LES separation, indicating a hiatal hernia. Following device implantation (**B**) the topographic plot shows an elevated intrabolus pressure (iBP) and integrated relaxation pressure (IRP) and a compensatory increase in esophageal body contractility

Persistent post-operative dysphagia, refractory to diet and multiple dilations, is a complex multi-faceted problem involving esophageal motility, device size, host response to implant, and individual subjective perception of dysphagia, all of which warrant further esophageal testing [23]. Endoscopy may reveal normal resistance at the EGJ, but marked resistance may indicate the formation of robust scar tissue surrounding the EGJ. Stasis esophagitis or candidiasis are possible as well. Barium swallow may reveal EGJ narrowing, delayed transit, and an elevated contrast column. Following MSA, HRM commonly shows an elevated IRP, elevated intrabolus pressure, and a compensatory increase in esophageal body contractions **(**Fig. 2**)**. [24] However, in patients with debilitating post-operative dysphagia, these findings are often more pronounced and HRM can reveal impaired peristaltic progression with poor bolus clearance. There have even been reports of pseudoachalasia following MSA from this increase in the GEJ outflow resistance [25].

**Fig. 3:**
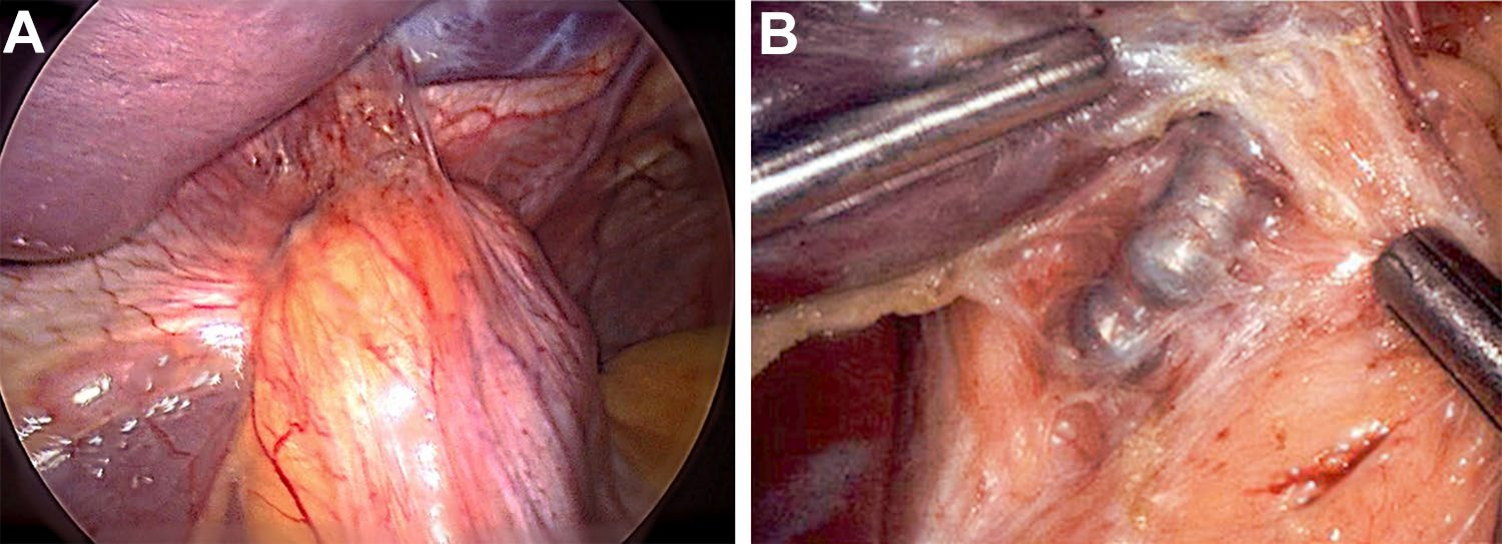
Dense fibrotic capsule surrounding the MSA. (**A**) Fibrosis completely encapsulated the titanium beads. (**B**) The capsule can be thick and can hold substantial tension even after partial opening

In the removal for dysphagia group, the post-implant improvements in heartburn, regurgitation and pH-normalization persisted after device removal, and were unaffected by additional ARS, suggesting that removal alone is adequate. Tatum et al. found similar results in their study of removal among 435 patients who had undergone MSA. They found that 77.8% of patients who required device removal for dysphagia did not require additional ARS. [10] These findings suggest that device removal does not return EGJ pressure to baseline; some degree of LES augmentation remains. The reason for this residual augmentation is likely due to the tissue reaction to the titanium beads of the MSA device. Studies of orthopedic and dental implants have demonstrated that titanium biologic implants stimulate a foreign body response, which walls off the titanium with a thick fibrous capsule [26–29]. Our experience with device removal demonstrated that a similar fibrous capsule forms around the gastroesophageal junction (Fig. 3). Initially, MSA works by increasing external augmentation at the LES with circumferential titanium magnets. However, as the capsule forms around the device, it further augments the barrier function of the lower esophageal sphincter. [30] When a MSA device is removed, the capsule remains, and the forces exerted by it can provide enough residual sphincter augmentation to control reflux and balance the EGJ resistance.

The addition of an anti-reflux surgery at the time of removal acts to counterbalance the expected decrease in EGJ resistance and increase in reflux caused by removing the augmentation. However, among those who underwent removal for dysphagia, the residual capsule appears to be enough to balance EGJ resistance, and additional anti-reflux surgery is unnecessary. By contrast when EGJ resistance after implantation was inadequate, as in our recurrent GERD group, removal alone resulted in failure to control symptoms, free patients from PPI use, or normalize distal esophageal pH. Therefore, additional ARS at the time of removal should be strongly recommended in these patients.

**Fig. 4:**
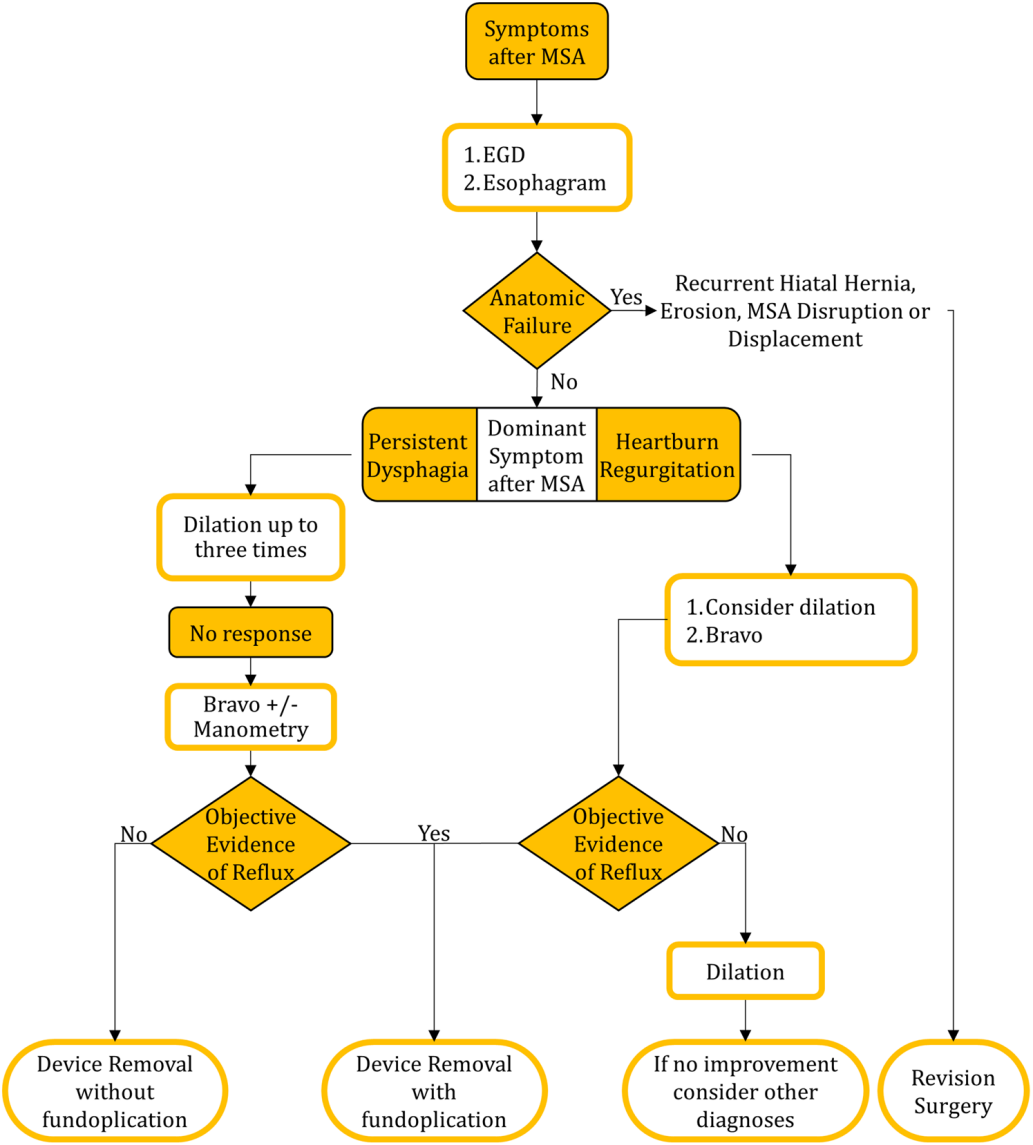
Proposed management paradigm for patients complaining of dysphagia or inadequate relief of their reflux symptoms after MSA

Almost half of patients who underwent device removal for persistent GERD symptoms reported dysphagia, and 29% of patients who underwent removal for dysphagia reported heartburn or regurgitation. Therefore, symptoms alone are insufficient to tailor surgical management. Patients being considered for device removal should undergo objective foregut evaluation. The assessment should begin with a revaluation of the postoperative anatomic integrity. A barium esophagram and EGD can identify recurrent hiatal hernia or device erosion, disruption, or displacement [31, 32]. In a symptomatic patient, any of these findings is a clear indication of failure and need for surgical revision [32, 33]. When the repair is anatomically intact endoscopic evaluation for esophagitis and ambulatory pH monitoring become essential. If worsening esophagitis or abnormal distal esophageal acid exposure are discovered, the initial augmentation has failed and additional ARS is needed. If a patient undergoing removal for dysphagia has also failed from a reflux control standpoint, the construction of a floppy Nissen or partial fundoplication should be considered. By contrast, in a patient with dysphagia with normal distal esophageal acid exposure, high IRP and poor bolus clearance on HRM can aid in identifying an elevated resistance at the GEJ [34, 35]. Based on the findings of the present study, device removal without additional ARS is adequate for these patients. Sustained non-dysphagia reflux symptoms despite unremarkable objective results can be due to a number of conditions including: non-acidic reflux, esophageal hypersensitivity, and sensations secondary to esophageal wall distension. Objective tests prior to device removal are crucial as the comparison to preoperative values, and the correlation of symptoms with objective findings, greatly assists with surgical decision-making and patient counseling. Therefore, we propose a treatment paradigm for the management of heartburn, regurgitation and/or dysphagia after MSA as demonstrated in Fig. 4.

Limitations of this study include its retrospective nature, small sample size, and variable post-implant and post-removal workup. Not all the patients underwent pH monitoring and manometry following device implant and removal. Further studies with larger populations and standardized objective testing regimens are necessary to confirm our findings.

## Conclusion

We found that device removal after MSA for persistent dysphagia or insufficient reflux control is necessary in 5.1% of implantations. Patients who had undergone removal for dysphagia had excellent symptom resolution and objective pH normalization, regardless of whether they had additional anti-reflux surgery at the time of removal. By contrast, patients who refused ARS at time of device removal for insufficient reflux control had significantly worse outcomes on all measures. Objective testing prior to device removal is necessary to identify the underlying mechanism MSA failure, and surgical management of these patients should be tailored to the indication for device removal.
